# Practical Points in the Home Treatment of Tuberculosis
*A paper read before the South Somerset Medical Club on September 17th, 1953.


**Published:** 1954-07

**Authors:** C. de W. Kitcat

**Affiliations:** Consultant Chest Physician, South Somerset


### PRACTICAL POINTS IN THE HOME TREATMENT OF TUBERCULOSIS*

BY

C. de W. KITCAT, M.R.C.S., L.R.C.P.

*Consultant Chest Physician, South Somerset*

In the past most patients with tuberculosis requiring treatment were admitted to a sanatorium. In recent years, however, bed shortage and new drugs have led to treatment being given by general physicians or surgeons and the family doctor. This is especially so in " closed " cases of tuberculosis, such as cervical adenitis, peritonitis, and genito-urinary tuberculosis. However, lack of experience with the chemotherapy of tuberculosis sometimes leads to the incorrect use of the drugs. Most cases of tuberculosis sooner or later come to the notice of the tuberculosis chest physicians and it is not uncommon for them to find that a patient has been given prolonged streptomycin alone, and that this may have made the tubercle bacilli resistant to streptomycin.

My first point, therefore, is a plea for co-operation between all branches of the profession in the treatment of tubercular conditions. I am not for one moment suggesting that the tuberculosis chest physician is the only person who should give treatment. I am, however, very sure that any chest physician would be very glad to co-operate in the planning of treatment, for it seems likely that the amount of the new drugs which can be given to any one patient in his life-time without his tubercle bacilli becoming resistant to them is strictly limited. It is, therefore, of the utmost importance to use judgment and experience in the timing, length and dosage of courses of chemotherapy.

### DRUG THERAPY

It is essential to adhere strictly to the Medical Research Council's recommendation that streptomycin, P.A.S. and isoniazid should only be given in combination and never singly.

*Streptomycin* is given nowadays usually as the sulphate or calcium chloride solution. Injections are given intramuscularly, the commonest site being the upper and outer quadrant of the buttocks. It is important to see that nurses appreciate this, for I have seen considerable sciatic pain resulting from an unfortunately placed injection. The dilution of the drug is also important if injections are to be painless. One gramme of streptomycin will dissolve in 1 c.c. of distilled water but it will be found, however, that 4–5 c.c. of water will be much pleasanter for the patient. A short post-injection massage of the area also helps in this respect. Nurses giving streptomycin should be told to wear rubber gloves, for it is possible to develop a dermatitis from continual contact with the solution and once it has been acquired it can be very troublesome.

The dosage varies; one gramme a day may be given in certain types of case, and with these daily dosages the patient is best in hospital. For cases at home the American custom of giving one gramme twice or three times a week works very well and is less demanding upon the time of busy district nurses. The one gramme is given in one dose for there appears to be no advantage in dividing it. One great advantage of giving streptomycin twice or thrice weekly is that it takes six months or more to give 72 grammes; bed rest is still the sheet-anchor of treatment, and experience shows that it is much easier to keep patients contented in bed while they are having what is to them recognizable treatment. Dihydro-streptomycin has caused deafness and is no longer used.

*A paper read before the South Somerset Medical Club on September 17th, 1953.

It is important to record the evening temperature taken for five minutes in the mouth because toxic manifestations are frequently preceded by a gradual rise of temperature, particularly in the first three weeks of the injections. The commonest toxic developments are rise of temperature, headaches, malaise, and the appearance of a rash resembling either measles or scarlet fever and this has led, on more than one occasion, to a patient being sent into a fever hospital. Should rashes appear, all treatment should be stopped, anti-histamines given and lotion such as lotio calamine oleosum applied.

When the reaction has settled, tests should be carried out to find out whether the patient is hyper-sensitive to streptomycin or P.A.S. or to both. To do this, 0.5 gramme of streptomycin is first given by itself, and, if the patient is hyper-sensitive the symptoms of fever, itching of the skin or return of the rash will occur within a few hours. If there is no reaction the patient is tested with the full one gramme. When this reaction, if it occurs, has settled, the patient is given a reduced dose of P.A.S. such as 2 grammes. If no reaction occurs a full dose of 5 grammes of P.A.S. is given and repeated several times to make quite sure. Some of the patients with fever and rashes can be desensitized by giving minimal and increasing doses of streptomycin and P.A.S., but this procedure is probably better carried out in hospital. Other toxic symptoms are giddiness, deafness and blurring of vision and in these cases streptomycin should be abandoned completely.

Lastly, except when streptomycin is given for a few days and only for infections other than by the tubercle bacillus, it should *never* be given alone and always in combination with P.A.S., or isoniazid. A continued watch too should be kept upon the total number of grammes of streptomycin which have been given in a patient's life and the mistake avoided of giving too much too early. Granted that the combination with another drug, such as P.A.S., postpones the time when the tubercle bacilli will become streptomycin-resistant, it seems likely that by the time a patient has had 200 or so grammes that this may well have occurred and therefore a regular note of the streptomycin score to date is very helpful.

*P.A.S.* (*para-amino salicylic acid*) should not be given by itself but in combination with streptomycin or isoniazid. The dosage varies between 10 and 20 grammes a day and the Medical Research Council's investigations have shown that the larger dose is more protective against the development of streptomycin resistance than is the smaller one. On the other hand P.A.S. is rather unpleasant and is apt, particularly in women, to cause nausea, vomiting and looseness of the bowels sometimes amounting to severe diarrhoea. In consequence the intermediate doses of 12 or 15 grammes a day of P.A.S. are often employed. The drug can be given in solution, or in cachets, or in enteric coated tablets—the last in the hope that if the P.A.S. is not liberated in the stomach the nausea and vomiting will be prevented. Some patients prefer one form and some another, but all too frequently if the patient is very sensitive to P.A.S. he will not tolerate it in any form. The requisite dose should be given preferably four times a day after food, never on an empty stomach. For abdominal discomfort the N.F. Pulv. Mag. Trisilicate Co. given with or after the dose sometimes helps; opium in the form of *Tinct. Chlorof. et Morphine Co.* or *Tr. Opii.* may offset looseness of the bowels. There are many different proprietary forms of P.A.S. in cachets, tablets, etc., made up in different strengths so the doctor must be careful to prescribe the correct daily dose. In the home treatment of tubercle, P.A.S. is particularly useful in combination of isoniazid because injections are avoided.

*Isoniazid* (*Iso-nicotinic acid hydrazide or I.N.H.*) is the latest of the chemo-therapeutic products in common use. It should not be employed by itself. Various doses have been used but nowadays the common dose for most cases is 200 milligrammes daily, given as one 50-milligramme tablet four times a day. Various side effects and toxic symptoms such as constipation, difficulty in passing urine, dizziness, hyper-reflexia, twitchings and nervous symptoms have been described but in the dosage suggested they seem to be very rare. Advantages of isoniazid are the rapid development of a feeling of well-being, and an increase in appetite. From the Medical Research Council's investigations it seems that when using streptomycin in combination with isoniazid to prevent resistance to isoniazid developing, the streptomycin should be given daily and not two or three times a week. In the past it was recommended that isoniazid should be given for one month only but from the latest report of the Medical Research Council in the *B.M.J.* of November 7th, 1953, it now appears that isoniazid can be given for three months provided that it is combined with daily streptomycin or 20 grammes daily of P.A.S. In addition the result combined with P.A.S. over a period of three months appears to be as good as with streptomycin combined with either P.A.S. or isoniazid.

### RESULTS

The various combinations of these drugs produce their most striking results on recent and early lesions, and their least effect on cases of long-standing tuberculosis with much fibrosis. In general, too, cases of glandular tuberculosis seem to derive less benefit than lesions in other tissues. In advanced pulmonary tuberculosis, however, is always worth while giving short courses of the drugs as these reduce cough, sputum and fever considerably for a time and also have some psychological effect.

The methods of chemotherapy are not a complete cure of tuberculosis in themselves and bed rest for long periods remains as essential as ever. The majority of cases of pulmonary tuberculosis too, require artificial pneumothorax, pneumoperitoneum or a phrenic crush. Many cases also will require such major operations as thoracoplasty and lung resection. In recent years however chemotherapy and methods of lung relaxation have produced such startling improvement, even in some advanced cases, that major surgery has become possible. As a result of all this the death rate from tuberculosis has declined in a most satisfactory way.

It is of interest that with all the latest methods of treatment available, the patient nowadays spends a longer time in bed and under treatment than he did in previous years, and, whereas in the past six to nine months in a sanatorium was usual, the patient nowadays is often in hospital for one and a half years or even more. On the other hand the results are undoubtedly very much better and we feel that at long last we are really getting to grips with this troublesome and tragic disease.

### PREVENTION BY B.C.G. INOCULATION

B.C.G. is a live vaccine from a strain of tubercle bacillus isolated in 1902, which through repeated sub-culture became so attenuated that since 1930 it has never been virulent for man. Though incapable of causing tuberculosis, it causes production of antibodies and converts the tuberculin-negative subject to a tuberculin-positive one. Tuberculin-negative persons are much more likely than tuberculin-positive persons to develop tuberculosis when they come in contact with infectious cases. Millions of children all over the world have been given B.C.G. without harm and where given there has been a striking diminution in the number of cases of such previously fatal conditions as miliary tuberculosis and infectious tubercular meningitis.

In recent years in this country, B.C.G. has been given to tuberculin-negative medical students and nurses and it is recommended for the tuberculin-negative children of families where there has been a case of pulmonary tuberculosis. The newly born child of a tuberculous mother is removed at once and given B.C.G. and retained in a nursery until it has become tuberculin-positive—a matter of 6–8 weeks. When an infectious adult is removed to a sanatorium any children are left alone for a period of six weeks to give time for possible conversion from recent infection; the child is then tuberculin tested and if negative he is given B.C.G. Where the adult case has never been found to be infectious, B.C.G. inoculation of children can be carried out while they remain in the house with the adult case. To prevent unnecessary needle-pricks for the child, it is common to use tuberculin jelly for the first test and if this is negative to do a final 1-100 intradermal Mantoux before inoculation. Incidentally where B.C.G. has been given to a tuberculin-positive and active case the harm is usually limited to an unpleasant local reaction at the site of injection. In the normal tuberculin-negative case no malaise or general reaction results and after three weeks a bluish-red papule with an indurated base begins to appear. The local reaction reaches maturity about the sixth week after which it slowly regresses leaving no scar. Occasionally a small area in the centre of the papule may discharge serum or form a shallow ulcer but these heal rapidly with the application of P.A.S. powder.

The conversion to tuberculin positive lasts for about five years but if the child or adult gets occasional doses of tubercle bacilli from natural sources they may remain tuberculin positive indefinitely. However, it is usual to re-test the child annually and to consider revaccinating should reversion have occurred.

There would seem to be a strong case for giving B.C.G. to all tuberculin-negative school-leavers to protect them from tubercle in the 15 to 25 age group when they are starting their life's work, for it is then that they otherwise have the greatest risk of becoming infected.

